# The Influence of Health-Related Behavior Profiles on College Students’ Perceptions of COVID-19 Safety Measures

**DOI:** 10.1007/s10900-024-01342-9

**Published:** 2024-02-27

**Authors:** Faith Shank, Megan Korovich, Alexandra Nicoletti, DJ Angelone, Meredith C. Jones

**Affiliations:** Department of Psychology, Rowan University, 201 Mullica Hill Rd, Glassboro, NJ 08028 USA

**Keywords:** Alcohol use, Cannabis use, Sexual behaviors, College students, Pandemic

## Abstract

College students often engage in multiple health-related behaviors simultaneously which can lead to negative outcomes and further risky behaviors. During the COVID-19 pandemic, college students reported decreased condom use, increased solitary cannabis use, and increased alcohol consumption. This current study aimed to (1) identify profiles of health-related behaviors (i.e., alcohol consumption, cannabis use, and sexual behaviors), and (2) determine if these profiles would differ in engagement and perceived effectiveness of COVID-19 preventative measures. Participants were 273 college students from a large Northeastern U.S. public university who completed surveys about health-related behaviors during the 2021 academic year. We used a latent profile analysis to identify distinct subgroups of college students based on their engagement in health-related behaviors. Based on fit indices a three-profile solution showed the best fit: low (*N* = 196), moderate (*N* = 54), and high (*N* = 23). Two one-way ANOVAs examined whether profile membership predicted engagement and perceived effectiveness of COVID-19 safety measures. Participants in the low health-related behaviors profile engaged in preventative measures more than students in the other two profiles. However, profile membership did not predict perceived effectiveness of preventative behaviors. Taken together, our results indicate that college students reporting lower levels of health-related behaviors engage in more preventative measures during a pandemic. Understanding distinct health-related behaviors profiles among college students, and their links with COVID-preventative health-related behaviors, can inform prevention strategies.

**Supplementary Information:**

The online version contains supplementary material available at 10.1007/s10900-024-01342-9.

## Health-Related Behaviors in College Students

Young adults, particularly during the transition into college life, engage in a variety of health-related behaviors [1]. This transition, known as emerging adulthood, is marked by identity exploration and engagement in both novel social and physical environments, and therefore new behaviors [2, 3]. Many college students’ behaviors are not consistent with health recommendation guidelines [4].

Substance use is common among college students, with many reporting high rates of alcohol use [5, 6]. In particular, 71% of college students reported having ever used alcohol and 66% reported drinking alcohol in the past three months [7]. Many college students endorse taking part in alcohol consumption in high [8, 9]. Students who endorse alcohol consumption, 40% reported consuming five or more drinks once or twice in the last two weeks, with 15% reporting three or more instances of binge drinking [7]. On another note, cannabis use among college students is at an all-time high, with 55% of college students reported having ever used cannabis [10–12]. Of cannabis users, 19% are considered to use at a moderately risky level [7].

Sex is another behavior that college students explore. In fact, 61% of students report having engaged in oral sex, 56% report vaginal intercourse, and 16% report anal sex [7]. College students report a variety of risky sexual behaviors, including a lack of birth control or condom use. While most students report only one sexual partner in the past 12 months, 13% report having had two partners, and 20% endorse having three or more partners [7].

## Clustering of Health-Related Behaviors

Certain health-related behaviors such as physical inactivity, poor diet, smoking cigarettes, substance use, and risky sexual behaviors can result in negative outcomes [13]. These behaviors tend to cluster, or co-occur, within individuals. The presence of numerous health-related behaviors may increase one’s vulnerability to chronic illness and premature mortality [14] over the presence of just one behavior [15]. Clustered behaviors are influential on one’s engagement in other health-related behaviors, which can increase one’s risk for unhealthy outcomes.

Behaviors associated with negative consequences tend to cluster among college students such as alcohol use, cannabis use, and sexual behaviors. For instance, students who endorse heavy drinking often also engage in more frequent risky sexual behaviors [17, 19]. In fact, up to a third of college students consume alcohol prior to engaging in sex, reporting an average of five drinks prior to engaging in sexual behavior [20]. Consuming alcohol prior to engaging in sex is also associated with higher rates of unprotected sex, particularly with casual partners [20].

## COVID-19 and Influence on Health- Related Behaviors

The COVID-19 pandemic has had unique influences on the engagement of health-related behaviors in college students as university closures disrupted college students’ daily lives. Most students had to move home, attend courses virtually, and practice social distancing, which affected interpersonal connections and stress levels and may have changed their engagement in various health-related behaviors [21, 22].

### Sexual Behavior

Though rates of sexual behavior remained consistent with 79% of college students engaging in sexual activity [23], there is mixed evidence regarding the impact of COVID-19 on sexual behaviors. Some college students reported a decrease in sexual activity [22], possibly due to lack of access to potential partners or concerns about virus transmission. During the pandemic, many reported a lower sexual risk perception [23]. College students reported little concern about contracting COVID-19 through sexual behaviors.

Additionally, college students were seen to engage in less frequent condom use and HIV/STI testing throughout the pandemic [22]. Only 25% of college students endorsed consistent condom use in the previous 3 months [22]. Decreased testing is potentially related to fewer sexual risk behaviors, lower perceived risk, or less accessibility to testing services.

### Alcohol Use

In addition to a change in sexual behaviors during the pandemic, many individuals changed their drinking habits, although the evidence is mixed [21, 24–26]. For college students, university closures were positively associated with higher frequency and consumption of alcohol in relation to their drinking behaviors pre-pandemic [27, 28]. Nearly 25% of college students reported increasing their alcohol consumption [22]; however, those who reported higher amounts of consumption were engaging in higher levels of consumption pre-pandemic when compared to peers [25, 29]. College students who were diagnosed with COVID-19, often experienced alcohol cravings, resulting in more frequent drinking behaviors [34].

On the other hand, evidence shows decreases in drinking patterns, potentially due to the lack of access to sources of alcohol, and reduced social interactions [21, 31]. College students drinking patterns are heavily associated with their peer networks and having others to engage with socially while drinking [32, 33]. Thus, during the pandemic, many students reported drinking more often with their families, instead of their peers [30]. These students were not; however, increasing the amount of alcohol consumed. In addition, there is evidence to suggest that those who had a higher perceived risk of COVID-19 consumed less alcohol [35].

### Cannabis Use

Overall, cannabis use was observed to decrease during the pandemic [37]. However, college students who were established as persistent cannabis users pre-pandemic maintained the frequency of their use throughout [37]. Further, social changes brought about by COVID-19 are thought to have increased the level of solitary cannabis use [38]. During the pandemic, 41% of college students who engage in solitary cannabis use reported using alone on at least 3 occasions weekly. Interestingly, college students who reported utilizing alcohol as a way to cope during the COVID-19 pandemic were also seen to engage in more frequent cannabis use [36].

### Engagement of Preventative Measures During COVID-19

While many students endorsed taking COVID-19 preventative measures seriously, others sought to continue attending social gatherings such as going to the bars [39]. Preventative measures (e.g., washing hands, social distancing), declined among college students throughout the pandemic, placing themselves at higher risk for contracting COVID-19. There appears to be a clustering of COVID preventative behaviors for college students such that students who engaged in social distancing also isolated themselves, including attending fewer social gatherings, visiting fewer stores and businesses [40]. COVID preventative behaviors are associated with lifestyle changes during COVID. For example, students who increased their smoking and drinking behaviors during the pandemic engaged in fewer preventative behaviors for COVID [41].

## Present Study

Little is known about how these health-related behaviors (i.e., alcohol use, cannabis use, sexual behavior) cluster among college students during the COVID-19 pandemic. The primary aim of this study was to examine how health-related behaviors cluster during the COVID-19 pandemic within a college population. Given the empirical support regarding the clustering of health behaviors [18], we predicted that these health-related behaviors would cluster during the pandemic. Our second aim was to determine whether profiles linked to clusters of health-related behaviors predict which college students are at higher risk of not engaging in COVID-19 preventative practices. These profiles may indicate the need for targeted interventions for college students who are less likely to engage in preventative practices for infectious diseases.

## Methods

### Participants and Procedures

Participants were 273 college students from a large public university in the Northeastern United States who took part in a larger study about campus-specific norms, drinking behaviors, and other risk behaviors between September 2021 and May 2022. Participants were recruited through a stratified random sample from a list of students provided to the researchers from the university’s registrar’s office. Eligibility criteria required that participants be between 18 and 26 years old and currently registered as a student at the university. Potential participants were invited through email and provided an anonymous link to participate if interested. All participants provided informed consent before beginning the study, and the larger survey took approximately one hour to complete. Those who completed the full survey were compensated $20.

Participants in our sample self-identified as 59.7% female (*n* = 163), 37.4% male (*n* = 102), and 5.2% transgender, genderqueer, or other (*n* = 14). The majority were White (74.0%, *n* = 202), followed by Asian (12.1%, *n* = 33), Black (5.5%, *n* = 15), and Native American or Native Alaskan (1.5%, *n* = 4); 6.2% did not identify with any of the provided groups and selected “other” (*n* = 17). Thirty-nine participants (14.3%) identified as Hispanic or Latino, and 11.0% (*n* = 30) considered themselves to be multiracial. Most participants were between 20 and 22 years old (68.0%, *n* = 184), with a mean age of 21.2 years.

Relatedly, the majority of participants were juniors or seniors (68.9%, *n* = 188) as opposed to first-years and sophomores (17.9%, *n* = 49). Eighty-six (31.5%) participants reported being first generation college students. In regard to housing, 36.8% lived with family (*n* = 99), 31.9% lived on-campus (*n* = 87), 30% lived off campus in a house or apartment (*n* = 82), and two participants lived in fraternity or sorority housing (0.7%). Most participants were “single,” defined as single and exclusively dating (44%, *n* = 120), single and not dating (39.2%, *n* = 107), or single and casually dating (12.5%, *n* = 34); Seven participants were married or had a life partner (2.6%), and five were engaged (1.8%).

### Measures

#### Demographics

Participants demographic profiles were generated with items assessing gender identity, age, race and ethnicity, year in college, living situation (e.g., dorm living, off-campus housing), Greek affiliation, and body weight and height (for BAC and other health-related determinations).

#### Drinking Patterns Questionnaire

Alcohol consumption was evaluated with numerous items to establish patterns of drinking. Participants were asked to report the average number of drinks they consumed on each day of the week on a 7-day grid modeled after the Daily Drinking Questionnaire [42]. They were also given items assessing the highest number of drinks they would consume on a given day, and within what time period, in order to gain a BAC estimation. Lastly, participants were asked the frequency of days where they drank more heavily (i.e., ≥ 4 drinks for females, and ≥ 5 drinks for males). The number of drinks per week consisted of the sum of the drinks that participants reported imbibing on each day of the week.

#### Cannabis Assessment

Participant cannabis use was determined by the Marijuana Questionnaire [43, 44]. The items on this scale refer to all forms of cannabis except for synthetic cannabis (e.g., Spice or K2). Items ask participants questions such as their age of first use and lifetime frequency of use. They were also asked to report their preferred use methods and settings in which they use cannabis (i.e., in social or private settings). Frequency and amount of cannabis use was assessed by asking in a typical session, day, or week, how much cannabis is consumed (in grams), and then by how many sessions participants have on a weekday versus the weekend. Average amount of cannabis used per week was used as the measurement for cannabis use in this study.

#### Modified AIDS Risk Behavior Assessment (ARBA)

Sexual behaviors were measured with a modified version of the AIDS Risk Behavior Assessment (ARBA) [45]. All items referred to situations where participants engaged in consensual sexual activity. Participants were asked about frequency of oral and penetrative sex within the past 3 months, current contraceptive use, and future condom use. They were also asked about STD and HIV testing, previous positive tests, and frequency of utilizing drugs or alcohol before sexual activity. Sexual behavior was calculated by summing the number of sexual partners in the past three months and adding one point each for those who had sex while using drugs/alcohol and without a condom.

#### Imperial College Report COVID-19 Questions

Participants were asked to complete a 14-item scale intended to assess an individual’s protective factors against COVID-19, their reasoning for changes in behavior, how they were obtaining information about COVID-19, and their perceptions of the effectiveness of certain preventive measures. This instrument is based on a measure of community perception of risk, exposure to related information, and preventative measures for COVID-19 [46].

### Statistical Analysis Plan

To begin, we examined the descriptive statistics of all the relevant variables of interest. Next, we identified the optimal number of profiles that fit our data via a latent profile analysis (LPA). An LPA identified latent subgroups of the data based on a certain set of variables. Next, we examined the characteristics of the final profiles. Lastly, we identified if the various profiles predicted engagement and perceived effectiveness of COVID-19 preventative measures.

#### Identifying the Optimal Latent Profile Solution

We considered models with one to five profiles. Model fit was assessed by examining the Akaike Information Criterion (AIC), the Bayesian Information Criterion (BIC), the Entropy, and average latent profile probabilities for most likely latent profile membership. Models with lower AIC, and BIC indicate a better fitting model [47, 48]. Higher entropy values suggest that the participants were correctly classified [49]. The latent profile probabilities indicated the probability an individual belongs to a specific profile versus another. For example, having the probability of 95%, suggests that a given person has a 95% probability of belonging to profile 1 versus belonging to profile 2 [50]. The model with the highest probability should be favored [50].

## Results

### Sample Descriptive Statistics

#### Alcohol Use

Participants in the sample drank, on average, 3.3 drinks per week (*SD* = 4.3), which ranged from 0 to 18 drinks per week. Participants drank around two to three times a month, and on a typical occasion would consume 3 drinks (*SD* = 3.8).

#### Cannabis Use

Approximately 44.0% of participants reported cannabis use. On average, participants used 0.90 gram of cannabis per week (*SD* = 2.7). Participants’ use of cannabis ranged from 0 to 24 g per week. The most frequent place participants used included: at a friend’s home (36.3%), home (30.8%), and at a party (25.6%).

#### Risky Sexual Behaviors

On average, participants scored a 1.7 for risky sexual behavior (*SD* = 1.7). 48% of participants reported having had oral sex with at least one person in the last month, and 42.7% reported having had vaginal or anal sex with at least one person in the last month. In addition, 52.0% of participants reported they have never used drugs or alcohol before sex, and 41.0% of participants reported not using a condom the last time they had sex.

#### COVID-19 Measures

Participants, on average, reported engaging in 4.4 (out of 7) preventative measures for COVID-19 (*SD =* 2.1). On average, participants reported that COVID-19 preventative measures were fairly effective to very effective (*M* = 2.4, *SD* = 0.4).

### Determination of Number of Profiles

Models containing 1-to-5 profile solutions based on fit indices were compared (Table 1). Based on the results, it was suggested that the 3-profile solution was optimal. This 3-profile solution showed the lowest AIC and BIC, highest entropy and highest average latent profile probabilities of the 1-to-5 profiles.

**Table 1:** Model fit statistics for 1 to 5 latent profiles

				Latent Profile Probabilities	
No. of profiles	AIC	BIC	Entropy	Minimum	Maximum
1	2333.216	2354.873	1.000	1.000	1.000
2	2217.789	2253.884	0.916	0.897	0.990
**3**	**2163.066**	**2213.599**	**0.909**	**0.878**	**0.983**
4	2171.068	2236.039	0.574	0.000	0.976
5	1973.166	2052.574	0.637	0.000	0.999

*Note* Bolded row indicated the chosen profile solution; AIC = Akaike’s Information Criterion; BIC = Bayesian Information Criterion

### Latent Profile Characteristics

**Fig. 1:**
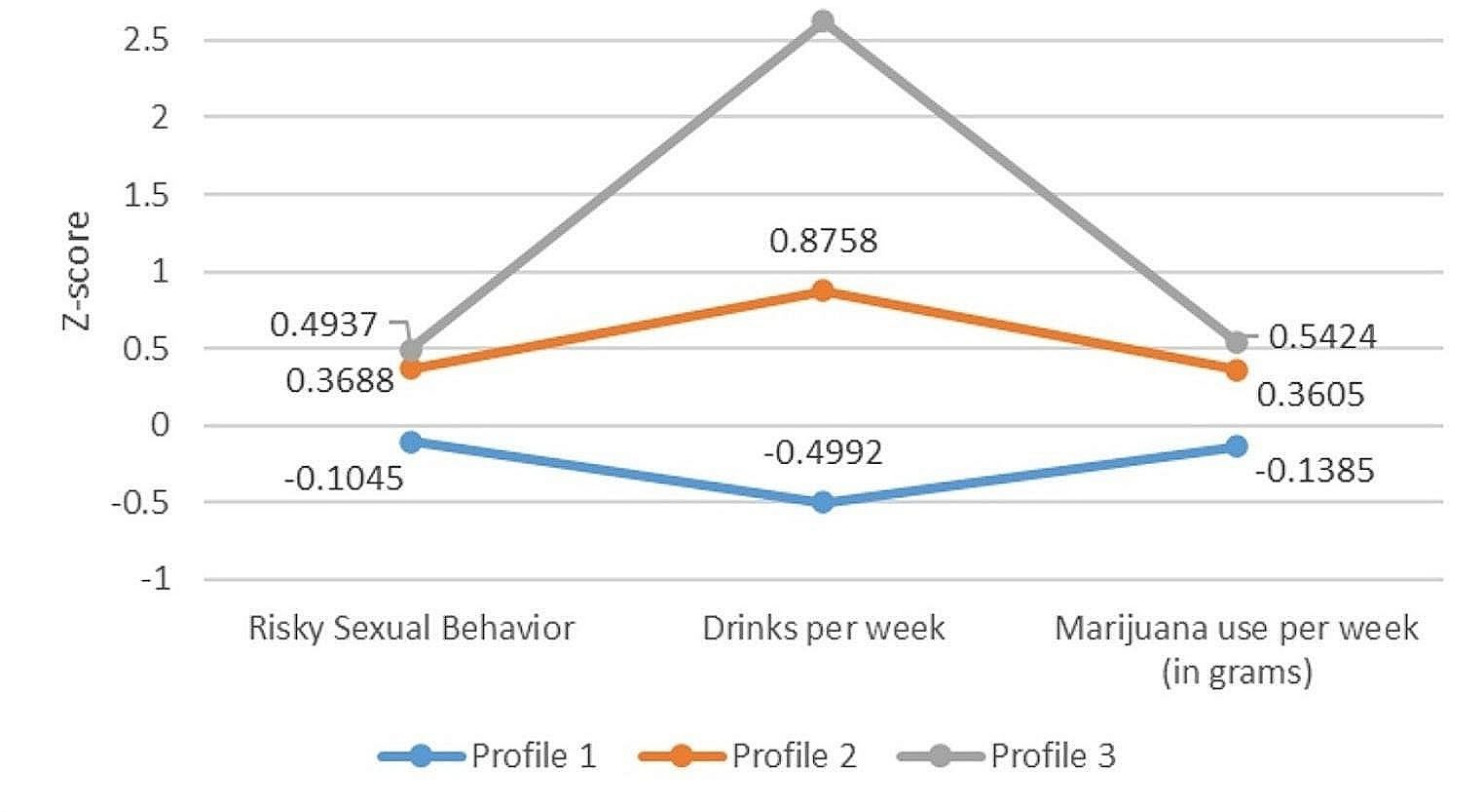
Scores on each unhealthy behavior dependent on membership in the latent profile for the 3-profile solution

The final chosen model was the 3-profile solution, which revealed three distinct and interpretable classes. There were distinguishable indicator response patterns for all three of the latent profiles, as shown by Fig. 1; Table 2. ***Profile 1*** (low engagement in unhealthy behaviors; 72.0%) represented a group of students who showed below average engagement in unhealthy behaviors. Specifically, students in this profile were likely to report drinking 0.5 SD below the sample mean, using cannabis 0.2 SD below the sample mean, and engaging in sexually risky behaviors 0.1 SD below the sample mean. In summary, participants in this profile engaged in unhealthy behaviors ranging from 0.1 to 0.5 SD below the sample mean. ***Profile 2*** (moderate engagement in unhealthy behaviors; 20.0%) a group of students who showed slightly above average engagement in unhealthy behaviors. Specifically, students in this profile were likely to report drinking 0.8 SD above the sample mean, using cannabis 0.3 SD above the sample mean, and engaging in sexually risky behaviors 0.3 SD above the sample mean. In summary, participants in this profile engaged in unhealthy behaviors about 0.3 SD above the sample mean. ***Profile 3*** (high engagement in unhealthy behaviors; 8.0%) represented a group of students who showed above average engagement in unhealthy behaviors. Specifically, students in this profile were likely to report drinking 2.5 SD above the sample mean, using cannabis 0.5 SD above the sample mean, and engaging in sexually risky behaviors 0.4 SD above the sample mean. All of which are significantly higher than participants mean engagement in profile 1. In summary, participants in this profile engaged in unhealthy behaviors ranging from 0.4 to 2.5 SDs above the sample mean.

**Table 2:** Descriptive statistics of unhealthy behaviors dependent upon membership in the 3-profile solution

Class	N	Sexual Risky Behavior		Drinks per week		Cannabis use per week (in grams)	
		M	SD	M	SD	M	SD
1	196	1.50	1.61	1.01	1.38	0.50	1.91
2	54	2.22	1.36	6.80	1.53	1.79	4.37
3	23	2.41	2.35	14.17	2.82	2.26	2.96

### Profile Membership Predicting COVID-19 Preventative Measures

After identifying three distinct profiles, we conducted two one-way ANOVAs to examine the aims of the study. First, a one-way ANOVA was conducted to examine how profile membership predicted engagement in COVID-19 preventative behaviors. The ANOVA results showed a significant effect of profile membership on preventative behaviors, *F*(2, 270) = 7.01, *p* < 0.001, eta^2^ = 0.05, indicating a medium effect size. Tukey’s post hoc analyses were used to examine significant differences between profile types. Results revealed significant differences between the profiles. Profile 2 (M = 3.8, SD = 2.1) engaged in significantly more preventative measures than profile 3 (M = 3.4, SD = 2.1), *p* < 0.05. Profile 1 (M = 4.7, SD = 2.0) engaged in significantly more preventative measures than profile 3 (M = 3.4, SD = 2.1), *p* < 0.05. There was no significant difference between profile 1 and profile 2.

Next, a one-way ANOVA was conducted to examine how profile membership predicted perceived effectiveness of COVID-19 preventative behaviors. The ANOVA results showed a significant effect of profile membership on preventative behaviors, *F*(2, 270) = 3.83, *p* = 0.023, eta^2^ = 0.03, indicating a small effect size. Although the overall *F* statistic was significant, the Tukey’s post hoc analyses revealed no significant differences between profile 1 (M = 2.4, SD = 0.4), profile 2 (M = 2.3, SD = 0.5), or profile 3 (M = 2.2, SD = 0.5) in perceived effectiveness of COVID-19 preventative behaviors.

## Discussion

The first aim of this study was to examine college students’ engagement in health-related behaviors and determine whether health-related behaviors clustered during COVID-19. Our sample reported, on average, drinking 3 drinks per week. This is inconsistent with previous findings, that during COVID-19 college students reported drinking, on average, approximately 9 drinks per week [51]. One explanation for this is that our sample of college students are low-risk drinkers and show more consistent low drinking behaviors. Their drinking patterns demonstrate a lack of heavy episodic drinking, imbibing around 4 drinks per week during the pandemic [52]. In addition, participants reported using 0.90 g of cannabis per week, which may be explained by a found decrease in cannabis use during COVID-19 [53]. Finally, our sample had consistent reporting of engagement in oral sex (48%), vaginal sex (43%), and not using a condom during intercourse (42%), when compared to other findings during COVID-19 [54].

We identified three latent profiles which distinguished between subgroups of participants based on their engagement in each of the health-related behaviors. Most participants in the sample belonged to profile 1, those who showed low engagement in the health risk behaviors. Only a small percentage of the sample reported high engagement in health risk behaviors (profile 3), and 20% reported moderate engagement. In general, we found that the level of engagement in health-related behaviors clusters for college students. In other words, having a high engagement in one health-related behavior makes one more likely to engage in other health-related behaviors similarly, which is consistent with the notion that these behaviors cluster together [1].

The second aim of the study was to examine if the latent profiles predicted engagement and perceived effectiveness in COVID-19 preventative measures. We found those who showed low engagement in the health risk behaviors reported the most engagement in preventative measures, which was significantly different than the other two profiles. Consistently, those who showed high engagement in the health risk behaviors reported the least amount of engagement in preventive measures. These findings suggest that college students who engage in more drinking, cannabis use, and sexual activity are less likely to engage in COVID-19 preventative strategies, thus are at a greater risk of contracting COVID-19. Thus, future interventions to increase engagement in COVID-19 preventative measures should target individuals who engage in high patterns of health-related behaviors overall. Our findings are also consistent with previous findings that those who engage in high levels of alcohol consumption and have more sexual partners are less likely to engage in health preventative measures [55].

On the other hand, our results revealed that engagement in health-related behaviors did not predict one’s perceived effectiveness of COVID-19 preventative measures. Taken together, college students perceive COVID-19 preventative measures as effective; however, those who engage in the health-related behaviors studied here are more likely to engage in the preventative measures. One explanation is that those who report high engagement in these health-related behaviors have low perceived susceptibility of contracting COVID-19, which aligns with engaging in health-related behaviors that may put them at additional risk to adverse health concerns [14, 16, 17]. Hence, future research needs to determine how to increase engagement in their engagement in preventative measures, for those who perceive these behaviors as effective.

While this study provides valuable findings, several limitations should be noted. First, the majority of participants were white, and juniors or seniors in college. This limits the generalizability of our findings to other college students from more diverse racial and ethnic backgrounds and to the population of the university as a whole. Future research should aim to examine if our findings are similar for more culturally diverse samples of college students. Second, this study focused on self-report measures for health-related behaviors, which limits the reliability and validity of the participants responses. Third, the measures used to assess the health-related behaviors are not on similar scales. Specifically, alcohol use is based upon drinks per week, while cannabis is based upon how much participants used in a typical week, and sexual behavior is based on frequency of partners in the past 3 months, not using a condom, and utilizing drugs or alcohol before sexual activity. Future research should focus on using more comprehensive and comparable assessments of these health-related behaviors. Lastly, as this study only focused on alcohol use, cannabis use, and sexual behaviors, our findings may not generalize to college students’ engagement in other types of health-related behaviors (e.g., diet, exercise, sleep).

Despite these limitations, our findings confirm that the clustering of health-related behaviors are consistent throughout COVID-19, even with changes in the engagement in these health-related behaviors. Finding that those who engage in these behaviors more frequently engage in less COVID-19 preventative measures can be used to better inform future interventions aiming to increase college students’ engagement in preventative-health behaviors. For instance, to prevent the spread of sicknesses throughout a university, providing education about preventative measures and their effectiveness may be beneficial, especially for students who tend to engage in these health-related behaviors. Future research should examine how the clustering of more health-related behaviors (e.g., sleeping, tobacco use, nutrition) influence engagement in preventive strategies to further understand which college students may benefit from further education.

### Electronic Supplementary Material

Below is the link to the electronic supplementary material.

Supplementary Material 1
