# Supplementary material for: The Influence of Health-Related Behavior Profiles on College Students’ Perceptions of COVID-19 Safety Measures
Source: J Community Health. 2024 Feb 27;49(4):1–9. doi: 10.1007/s10900-024-01342-9 (PMC11306267; doi:10.1007/s10900-024-01342-9)
Supplement: Supplementary file 1 — Supplementary Material 1 [file 10900_2024_1342_MOESM1_ESM.docx]

February 9^th^, 2024

Dear Editor,

Enclosed find the revised version of *The influence of health-related behavior profiles on college students’ perceptions of COVID-19 safety measures* (JOHE-D-23-01810R1). We have carefully considered your comments and have revised the manuscript accordingly. We are pleased by the reviewers’ assessment of our work, and we found their suggestions to be most helpful in improving our manuscript. We hope you find our responses suitable. Below we provide a point-by-point summary of how we addressed the issues raised.

**Editor’s comments**

COMMENTS FOR THE AUTHOR:

**Point 1.** “The introduction is ~4 pages; recommend reducing and moving some of the details to the discussion.”

**Reply.** We condensed the introduction, making it a total of 3 pages. Consistent with the reviewers suggestion, we moved some details from the introduction to the discussion.

**Point 2.** “In Methods, the Participants and Procedures subsection has study results, so those details should be moved to the Results section.”

**Reply.** There are no statistical analyses/results in the methods section. However, we do include descriptive information about our participants in the participants section.

**Point 3.** “The Methods section should have a description of the statistical methods that were applied.”

**Reply.** The methods section has a “Data Analysis Plan”, we renamed the section to the “Statistical Analysis Plan” to make this more clear.
